# Preoperative Inflammatory Markers as Predictors of Ovarian Tumor Malignancy: A Retrospective Diagnostic Study

**DOI:** 10.3390/medicina62081613

**Published:** 2026-08-21

**Authors:** Denis Gruber, Anca Mihaela Bina, Ahmed Abu-Awwad, Octavia Valentina Tinei, Simona-Alina Abu-Awwad, Cosmin Citu, Adrian Ratiu, Bianca Huidu, Sergiu Costescu, Zoran Laurentiu Popa

**Affiliations:** 1 Doctoral School, ‘Victor Babes’ University of Medicine and Pharmacy, 300041 Timisoara, Romaniaoctavia.tinei@umft.ro (O.V.T.);; 2 “Pius Brînzeu” Emergency Clinical County Hospital, Bld Liviu Rebreanu, No. 156, 300723 Timisoara, Romaniaahm.abuawwad@umft.ro (A.A.-A.); costescu.sergiu@umft.ro (S.C.);; 3 Centre for Translational Research and Systems Medicine, “Victor Babes” University of Medicine and Pharmacy, 300041 Timisoara, Romania; 4 Department of Obstetrics and Gynecology, “Victor Babes” University of Medicine and Pharmacy, 300041 Timisoara, Romania; citu.ioan@umft.ro; 5 Department XV-Discipline of Orthopedics-Traumatology, “Victor Babes” University of Medicine and Pharmacy, 300041 Timisoara, Romania; 6 Research Center University Professor Doctor Teodor Sora, “Victor Babes” University of Medicine and Pharmacy, 300041 Timisoara, Romania

**Keywords:** ovarian tumors, ovarian cancer, inflammatory markers, neutrophil-to-lymphocyte ratio, platelet-to-lymphocyte ratio, lymphocyte-to-monocyte ratio, risk stratification, diagnostic model

## Abstract

*Background and Objectives*: Ovarian cancer remains the most lethal gynecologic malignancy, largely due to late diagnosis and the limitations of currently available diagnostic tools. Systemic inflammatory markers derived from routine blood tests have emerged as potential low-cost biomarkers, reflecting the interaction between tumor biology and the host immune response. This study aimed to evaluate the association and diagnostic value of preoperative inflammatory markers in distinguishing malignant from non-malignant ovarian tumors. *Materials and Methods*: This retrospective, single-center observational study included 780 adult female patients diagnosed with ovarian tumors and treated surgically between January 2020 and December 2025. Preoperative hematological parameters were obtained from complete blood count analyses performed before any therapeutic intervention. The neutrophil-to-lymphocyte ratio (NLR), platelet-to-lymphocyte ratio (PLR), and lymphocyte-to-monocyte ratio (LMR) were calculated. Comparisons were performed among benign, borderline, and malignant tumors. To avoid collinearity between mathematically interrelated inflammatory indices, three separate multivariable logistic regression models were constructed, each including age, CA-125, and one inflammatory index. Diagnostic performance of the individual inflammatory indices was assessed using receiver operating characteristic (ROC) curve analysis. *Results*: Patients with malignant tumors were significantly older and exhibited higher NLR and PLR values and lower LMR values than patients with non-malignant tumors (all *p* < 0.001). In separate multivariable models adjusted for age and CA-125, higher NLR (OR = 1.79, 95% CI: 1.41–2.27, *p* < 0.001) and PLR (OR = 1.43, 95% CI: 1.17–1.75, *p* = 0.001) remained significantly associated with malignant ovarian tumors, whereas higher LMR was inversely associated with malignancy (OR = 0.66, 95% CI: 0.52–0.85, *p* = 0.001). ROC analysis demonstrated moderate discriminative ability for NLR and PLR (AUC = 0.72 for both), whereas LMR showed lower discriminative performance (AUC = 0.64). *Conclusions*: Preoperative systemic inflammatory indices are significantly associated with ovarian tumor malignancy. NLR and PLR were positively associated with malignant disease, whereas LMR showed an inverse association, and these relationships remained significant after adjustment for age and CA-125. However, their individual discriminative performance was modest, suggesting that these readily available inflammatory indices should be considered complementary rather than standalone biomarkers for preoperative risk assessment.

## 1. Introduction

Despite remarkable progress in oncologic care over recent decades, ovarian cancer continues to represent the most lethal gynecologic malignancy and remains the second most common gynecologic cancer in developed countries, following endometrial carcinoma [1,2]. Globally, it accounts for approximately 150,000 deaths each year, with epithelial ovarian cancer comprising the predominant histological subtype. A major contributor to this unfavorable prognosis is the silent and nonspecific clinical course of the disease, which results in nearly 70% of cases being diagnosed at advanced stages (FIGO III–IV) [3].

Over time, multiple diagnostic and prognostic strategies have been implemented in an attempt to improve early detection and guide therapeutic decision-making. These include imaging-based algorithms such as the IOTA models, serum biomarkers like CA-125, and composite indices including the ROMA score, alongside detailed histopathological evaluation. While these tools have undoubtedly enhanced diagnostic accuracy, their impact on long-term outcomes, such as overall survival (OS), progression-free survival (PFS), disease-free survival (DFS), and cancer-specific survival (CSS), remains suboptimal. In parallel, the increasing complexity of oncologic care, particularly with the advent of targeted therapies such as poly (ADP-ribose) polymerase inhibitors (PARPi), has further emphasized the need for more refined and individualized risk stratification [4,5].

In this evolving landscape, growing attention has been directed toward the role of systemic inflammation in cancer progression. Peripheral blood inflammatory markers (PBIMs) have emerged as attractive candidates, as they capture, in a simple and accessible manner, the dynamic interplay between tumor biology and host immune response [6]. Unlike traditional tumor markers, these indices are inexpensive, reproducible, and universally available, making them particularly valuable in resource-limited settings, where ovarian cancer continues to impose a disproportionate burden of mortality [6,7].

Among the most extensively investigated PBIMs are the neutrophil-to-lymphocyte ratio (NLR), platelet-to-lymphocyte ratio (PLR), monocyte-to-lymphocyte ratio (MLR), and lymphocyte-to-monocyte ratio (LMR), as well as composite indices such as the systemic immune-inflammation index (SII = platelet × neutrophil/lymphocyte) and the systemic inflammatory response index (SIRI = neutrophil × monocyte/lymphocyte). More recently, red cell distribution width (RDW) has also been explored as a potential marker of systemic inflammatory imbalance [8].

The biological rationale underlying these markers is deeply rooted in the concept of the tumor microenvironment, where a continuous and bidirectional interaction between malignant cells and host-derived inflammatory mediators drives tumor initiation, progression, and metastatic dissemination. This paradigm, first conceptualized in Stephen Paget’s “seed and soil” theory, remains a cornerstone of contemporary oncologic understanding [9,10].

Although widely used in clinical practice, traditional tumor markers such as CA-125 present important limitations, particularly in terms of specificity and accessibility. In contrast, systemic inflammatory indices offer a complementary perspective by reflecting the host’s immunological status. Among these, SII has shown promise, with several studies demonstrating its independent predictive value in ovarian malignancy, especially in scenarios where CA-125 alone may be insufficient [11,12].

From a prognostic standpoint, accumulating evidence consistently links elevated inflammatory markers, particularly NLR and PLR, to adverse clinical outcomes, including reduced OS and shorter progression-free intervals. Notably, higher threshold values appear to enhance their discriminative capacity, further supporting their potential role in risk stratification [13,14,15].

Taken together, these findings suggest that integrating systemic inflammatory markers into routine clinical assessment may provide additional, clinically meaningful insights into disease behavior, ultimately contributing to more personalized therapeutic strategies.

Although several previous studies have reported associations between inflammatory markers and ovarian malignancy, important limitations remain regarding the integration of these indices with established tumor biomarkers such as CA-125, the evaluation of histopathological heterogeneity, and the assessment of adjusted predictive models in real-world clinical cohorts. Furthermore, data directly comparing the complementary diagnostic contribution of inflammatory indices beyond conventional biomarkers remain relatively limited and inconsistent across different study populations [16,17,18].

Therefore, the aim of the present study was to evaluate the predictive value of selected routinely available inflammatory indices, specifically neutrophil-to-lymphocyte ratio (NLR), platelet-to-lymphocyte ratio (PLR), and lymphocyte-to-monocyte ratio (LMR), in distinguishing malignant from non-malignant ovarian tumors and to assess their potential complementary role alongside CA-125.

## 2. Materials and Methods

### 2.1. Study Design and Setting

This retrospective single-center observational study was conducted at the Gynecology Department of the “Pius Brînzeu” Emergency Clinical County Hospital, Timișoara, Romania, a tertiary referral center serving patients from Western Romania.

The study included consecutive adult female patients diagnosed with ovarian tumors and surgically treated between 1 January 2020 and 31 December 2025.

Although the analyzed cohort covered a predefined retrospective time interval, data extraction and database construction were initiated only after Ethics Committee approval was obtained on 10 November 2025. Following approval, clinical, laboratory, and histopathological data were collected from electronic medical records according to the predefined inclusion and exclusion criteria.

Additional eligible cases identified before the end of the study period were progressively incorporated into the study database in accordance with the approved protocol.

The retrospective design enabled the evaluation of routinely collected clinical and laboratory parameters in a large cohort of patients with ovarian tumors, reflecting real-world clinical practice.

All procedures related to data collection, processing, and analysis were conducted in accordance with institutional protocols and ethical standards, ensuring methodological rigor and the protection of patient confidentiality.

### 2.2. Inclusion and Exclusion Criteria

**Figure 1:**
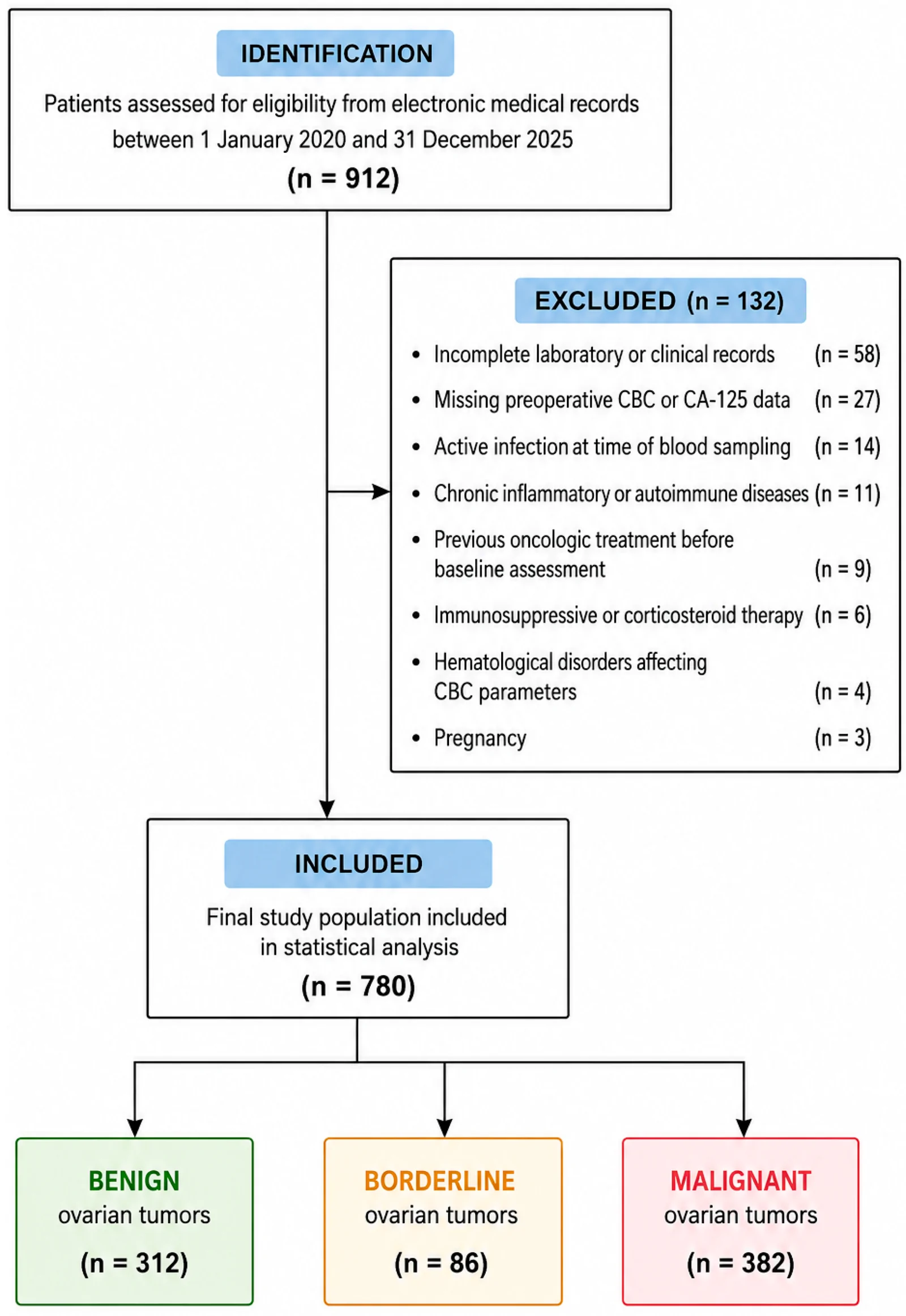
STROBE-compliant flowchart of patient selection and study population inclusion.

A total of 912 patients were initially assessed for eligibility, of whom 132 were excluded due to predefined clinical and laboratory criteria. The final study cohort consisted of 780 patients classified as benign (*n* = 312), borderline (*n* = 86), and malignant ovarian tumors (*n* = 382). The patient selection process and study eligibility criteria are summarized in Figure 1.

Adult female patients (≥18 years) diagnosed with an ovarian tumor [19] and surgically treated at the Gynecology Department during the predefined study period were considered eligible for inclusion.

Only patients with a definitive histopathological diagnosis of benign, borderline, or malignant ovarian tumors [20,21] were included to ensure diagnostic accuracy and uniform tumor classification across the study cohort.

To allow a reliable assessment of systemic inflammatory status, only patients with complete preoperative laboratory data were considered eligible. Specifically, complete blood count (CBC) analyses and serum CA-125 measurements performed at the time of diagnosis and prior to any therapeutic intervention (surgical, chemotherapeutic, corticosteroid, or anti-inflammatory treatment) [22] were required.

This criterion was considered essential to ensure that the calculated inflammatory indices, including neutrophil-to-lymphocyte ratio (NLR), platelet-to-lymphocyte ratio (PLR), and lymphocyte-to-monocyte ratio (LMR), accurately reflected the intrinsic interaction between tumor biology and host immune response rather than treatment-related alterations [23]. Complete blood count (CBC) and serum CA-125 measurements obtained within 3 days before surgery and prior to any therapeutic intervention were required for inclusion.

In addition, only patients with sufficiently detailed clinical and medical records were included, allowing the extraction of key demographic, laboratory, and histopathological variables necessary for statistical analysis and internal cohort consistency.

Patients were excluded if one or more of the following conditions were present:

• Active infections at the time of blood sampling [24,25].
• Chronic inflammatory or autoimmune diseases.
• Ongoing immunosuppressive therapy, corticosteroid treatment, or other medications known to significantly influence leukocyte dynamics.
• Previous oncologic treatment, including chemotherapy, radiotherapy, or recent major surgical interventions performed before baseline laboratory assessment.
• Hematological disorders affecting blood cell lines, such as leukemia or myelodysplastic syndromes.
• Pregnancy.
• Incomplete clinical, laboratory, or histopathological records.

These exclusion criteria were applied in order to minimize potential confounding factors that could independently influence systemic inflammatory markers and compromise the interpretation of tumor-related inflammatory profiles.

### 2.3. Data Collection

Data were retrospectively extracted from electronic medical records using a standardized data collection protocol designed to ensure consistency and minimize potential sources of bias.

The variables analyzed included demographic and clinical characteristics, such as age at diagnosis and area of residence (urban or rural). Tumor-related variables comprised histopathological subtype and tumor classification (benign, borderline, or malignant). Histopathological subtypes were additionally classified as epithelial and non-epithelial ovarian tumors in order to account for potential biological differences in systemic inflammatory profiles.

Preoperative laboratory parameters were obtained from blood samples collected within 3 days before surgery and prior to any therapeutic intervention. These included leukocyte, neutrophil, lymphocyte, monocyte, eosinophil, basophil, erythrocyte, and platelet counts. Serum CA-125 was measured within the same preoperative time window. The definitive histopathological diagnosis was established from the surgical specimen obtained during the corresponding operative procedure. These included leukocyte, neutrophil, lymphocyte, monocyte, eosinophil, basophil, erythrocyte, and platelet counts. Serum tumor marker levels (CA-125) were also recorded.

Based on these parameters, systemic inflammatory indices, namely the neutrophil-to-lymphocyte ratio (NLR), platelet-to-lymphocyte ratio (PLR), and lymphocyte-to-monocyte ratio (LMR), were calculated for each patient, reflecting the interplay between tumor-related inflammation and host immune response.

All data were anonymized prior to analysis and stored in a secure, password-protected database. Data integrity was ensured through systematic verification procedures performed before statistical processing.

FIGO staging data were not consistently available across all cases and were therefore not included in the present analysis.

### 2.4. Inflammatory Marker Calculation

To assess the role of systemic inflammation in ovarian tumor biology, three widely recognized hematologic indices were calculated using standard formulas derived from baseline CBC parameters.

The neutrophil-to-lymphocyte ratio (NLR) was defined as the ratio between absolute neutrophil and lymphocyte counts, serving as a surrogate marker of the balance between pro-inflammatory activity and adaptive immune response.

The platelet-to-lymphocyte ratio (PLR) was calculated by dividing the platelet count by the absolute lymphocyte count and has been associated with tumor progression, angiogenesis, and prothrombotic states.

The lymphocyte-to-monocyte ratio (LMR) was determined as the ratio between lymphocyte and monocyte counts and is generally interpreted as an indicator of host immune competence relative to tumor-promoting inflammatory activity.

All indices were derived from laboratory values obtained at diagnosis, prior to any therapeutic intervention. These variables were analyzed as continuous parameters. For multivariable analyses, each inflammatory index was evaluated in a separate regression model together with age and serum CA-125 to avoid the simultaneous inclusion of mathematically interrelated indices.

Although additional inflammatory indices such as SII, SIRI, RDW, and MLR were reviewed in the literature and considered during study conceptualization, the present analysis focused on NLR, PLR, and LMR because these markers are among the most consistently validated, easily reproducible, and clinically accessible inflammatory indices reported in ovarian tumor assessment. Furthermore, these indices demonstrated the most complete data availability across the retrospective cohort and were therefore selected for the final statistical analyses.

### 2.5. Statistical Analysis

Statistical analyses were performed using GraphPad Prism version 9.0 (GraphPad Software, San Diego, CA, USA) and IBM SPSS Statistics version 26 (IBM Corp., Armonk, NY, USA).

Continuous variables were expressed as mean ± standard deviation (SD) for normally distributed data and median with interquartile range (IQR) for non-normally distributed variables. Distribution normality was assessed using the Shapiro–Wilk test.

Between-group comparisons were performed using the independent-samples *t*-test or one-way ANOVA for normally distributed variables and the Mann–Whitney U test or Kruskal–Wallis test for non-parametric data, as appropriate.

To reduce the risk of type I error related to multiple comparisons, *p*-values derived from inflammatory marker analyses were adjusted using the Benjamini–Hochberg false discovery rate (FDR) correction. Adjusted *p*-values < 0.05 were considered statistically significant.

Multivariable binary logistic regression analyses were performed to evaluate the adjusted association between inflammatory indices and malignant ovarian tumors. Because NLR, PLR, and LMR are derived from overlapping hematological parameters and are therefore mathematically interrelated, they were not entered simultaneously into the same regression model. Instead, three separate multivariable logistic regression models were constructed. Model 1 included age, CA-125, and NLR; Model 2 included age, CA-125, and PLR; and Model 3 included age, CA-125, and LMR. CRP was evaluated descriptively as an additional marker of systemic inflammation but was not included in the predefined multivariable models or ROC analyses, which focused specifically on the CBC-derived inflammatory indices NLR, PLR, and LMR. Age and CA-125 were selected a priori as adjustment variables based on their established clinical relevance in the preoperative assessment of ovarian tumors and their availability across the study cohort. The modeling strategy was designed to evaluate whether each inflammatory index remained associated with malignancy after adjustment for these routinely available clinical and biochemical parameters, rather than to develop a comprehensive clinical prediction model. Other potentially relevant patient characteristics, including body mass index, smoking status, menopausal status, and comorbidities, were considered; however, these variables were not consistently documented across the retrospective cohort and were therefore not included in the predefined multivariable models.

The dependent variable was malignant ovarian tumor versus non-malignant tumor, with benign and borderline tumors combined in the reference category. This grouping was selected to address the primary diagnostic objective of distinguishing invasive ovarian malignancy from tumors without histologically confirmed invasive malignancy. Given that borderline ovarian tumors represent a distinct clinicopathological entity with different management implications from benign tumors, a sensitivity analysis excluding borderline tumors was additionally performed to assess whether their inclusion in the non-malignant reference group influenced the observed associations. In this sensitivity analysis, malignant tumors were compared exclusively with benign tumors.

Odds ratios (ORs) were calculated using standardized z-scores to allow comparability between variables. Results were reported with 95% confidence intervals (CIs). The 95% confidence level was selected to ensure consistency with the predefined two-sided significance threshold of α = 0.05. The Benjamini–Hochberg FDR procedure was used to address multiplicity within prespecified families of inflammatory-marker comparisons, whereas 95% CIs were used to quantify the precision of individual regression effect estimates. Model performance was characterized using the Hosmer–Lemeshow goodness-of-fit test and Nagelkerke’s R^2^.

Receiver operating characteristic (ROC) curve analysis was performed to evaluate the discriminative performance of each inflammatory marker individually. Optimal cutoff values for the individual inflammatory markers were determined using the Youden index. Supplementary analysis was additionally performed, in which the malignant group was restricted to the three epithelial ovarian carcinoma subtypes represented in the cohort (serous, endometrioid, and mucinous carcinomas) and was compared with the full non-malignant reference group comprising benign and borderline ovarian tumors.

All statistical tests were two-tailed, and statistical significance was defined as *p* < 0.05 after adjustment for multiple testing.

### 2.6. Ethical Considerations

The study was conducted in accordance with the Declaration of Helsinki and approved by the Local Ethics Committee of the “Pius Brînzeu” Emergency County Hospital, Timișoara (Decision No. 581/10 November 2025).

Ethics approval was obtained prior to the initiation of any data extraction procedures. Following approval, retrospective clinical data were collected from the medical records of patients treated during the predefined study interval (1 January 2020–31 December 2025), according to the approved study protocol.

Additional eligible retrospective cases identified before the end of the study period were progressively incorporated into the database.

Given the retrospective nature of the study and the use of fully anonymized data collected during routine clinical practice, the requirement for informed consent was waived by the institutional ethics committee.

## 3. Results

A total of 780 patients were included in the final analysis. The mean age of the cohort was 42.9 ± 15.0 years, with a median age of 42.0 years (IQR 31.0–52.0). Baseline hematological and inflammatory parameters are summarized in Table 1.

The median neutrophil-to-lymphocyte ratio (NLR) was 2.14 (IQR 1.49–3.09), while median platelet-to-lymphocyte ratio (PLR) and lymphocyte-to-monocyte ratio (LMR) values were 137.84 and 5.36, respectively. Overall, the cohort demonstrated a wide variability in inflammatory markers, reflecting the heterogeneity of ovarian tumor pathology.

Significant differences were observed across tumor groups in terms of both hematological and inflammatory parameters (Table 2).

Patients with malignant tumors were older compared to those with benign lesions (45.8 ± 15.4 vs. 39.8 ± 14.2 years, *p* < 0.001). A progressive increase in inflammatory markers was observed across groups, with the highest values recorded in malignant tumors.

Median NLR values increased from 2.12 in benign tumors to 3.01 in malignant cases (*p* < 0.001), while PLR showed a similar pattern, rising from 128.5 to 172.9 (*p* < 0.001). In contrast, LMR demonstrated an inverse trend, decreasing significantly from benign to malignant tumors (6.48 vs. 4.72, *p* < 0.001). CRP levels also differed significantly across tumor groups, with progressively higher values observed in malignant ovarian tumors compared with benign and borderline lesions (*p* < 0.001), further supporting the presence of an enhanced systemic inflammatory response in malignant disease.

**Figure 2:**
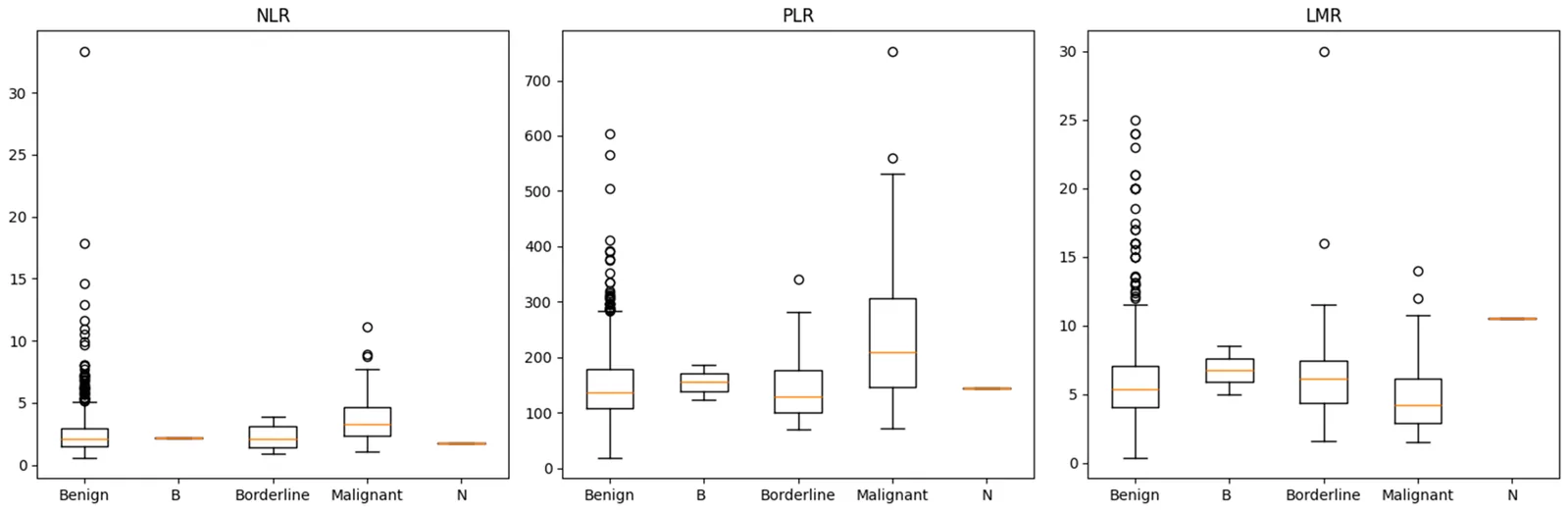
Distribution of systemic inflammatory markers according to tumor type. (**A**) Neutrophil-to-lymphocyte ratio (NLR), (**B**) platelet-to-lymphocyte ratio (PLR), and (**C**) lymphocyte-to-monocyte ratio (LMR) across benign, borderline, and malignant ovarian tumors. Boxplots represent the median and interquartile range (IQR), with whiskers indicating the range and individual points representing outliers.

These findings are visually illustrated in Figure 2, which highlights the clear separation between groups and the progressive shift in inflammatory profiles associated with malignancy.

The histopathological distribution of ovarian tumors demonstrated substantial biological heterogeneity across benign, borderline, and malignant lesions. 

Benign ovarian tumors included both epithelial and non-epithelial lesions, with epithelial cystic tumors representing the predominant subgroup. In contrast, all borderline and malignant ovarian tumors included in the present cohort belonged to the epithelial ovarian tumor category.

Among malignant ovarian tumors, serous carcinoma represented the predominant histopathological subtype, accounting for 47.6% of cases, followed by endometrioid carcinoma (42.9%) and mucinous carcinoma (9.5%).

Given the potential influence of histopathological subtype on systemic inflammatory response, a supplementary subgroup analysis restricted to epithelial ovarian carcinomas was additionally performed in order to evaluate the consistency of inflammatory marker associations within histologically related tumor entities (Table 3).

**Table 3:** Histopathological distribution of ovarian tumors.

Histopathological Subtype	Benign Tumors *n* (%)	Borderline Tumors *n* (%)	Malignant Tumors *n* (%)
Epithelial tumors	198 (63.5%)	86 (100%)	382 (100%)
Serous tumors	74 (23.7%)	48 (55.8%)	182 (47.6%)
Mucinous tumors	68 (21.8%)	38 (44.2%)	36 (9.5%)
Endometrioid tumors	24 (7.7%)	–	164 (42.9%)
Brenner tumors	32 (10.3%)	–	–
Non-epithelial benign tumors	114 (36.5%)	–	–
Mature teratoma	58 (18.6%)	–	–
Endometriotic cysts	31 (9.9%)	–	–
Fibroma/fibrothecoma	25 (8.0%)	–	–

Data are presented as number of cases and percentage within each tumor category. Borderline and malignant ovarian tumors consisted exclusively of epithelial ovarian neoplasms, while benign tumors demonstrated greater histopathological heterogeneity, including both epithelial and non-epithelial lesions.

Supplementary analysis was performed, in which the malignant group was restricted to the three epithelial ovarian carcinoma subtypes represented in the present cohort: serous, endometrioid, and mucinous carcinomas. This malignant epithelial group was compared with the full non-malignant reference group, comprising all benign and borderline ovarian tumors included in the study, irrespective of epithelial or non-epithelial histology. Thus, the analysis was restricted to epithelial histology only within the malignant group and should not be interpreted as a comparison exclusively between epithelial tumors. Higher NLR and PLR values and lower LMR values were observed in the malignant epithelial group compared with the non-malignant reference group (all *p* < 0.001), with similar differences observed for CA-125 levels (Table 4).

**Table 4:** Comparison of inflammatory indices between the malignant epithelial carcinoma group and the non-malignant ovarian tumor reference group.

Variable	Non-Malignant Tumors (Benign + Borderline)	Epithelial Ovarian Carcinomas	*p*-Value
NLR	2.19 (1.48–2.94)	3.08 (2.01–4.31)	<0.001
PLR	131.4 (104.2–168.5)	176.2 (138.7–225.9)	<0.001
LMR	6.31 (4.76–7.89)	4.68 (3.54–5.97)	<0.001
CA-125	22.1 (11.4–46.8)	194.6 (58.3–655.2)	<0.001

The malignant group comprised serous, endometrioid, and mucinous ovarian carcinomas. The non-malignant reference group comprised all benign and borderline ovarian tumors included in the study, including both epithelial and non-epithelial benign lesions.

Because NLR, PLR, and LMR are mathematically interrelated inflammatory indices derived from overlapping hematological parameters, they were not entered simultaneously into the same multivariable regression model. Instead, three separate multivariable logistic regression models were constructed to evaluate the adjusted association of each inflammatory index with ovarian malignancy. Each model included age and serum CA-125 as covariates together with one inflammatory marker (NLR, PLR, or LMR). The results are presented in Table 5.

To assess whether the inclusion of borderline tumors in the non-malignant reference category influenced the observed associations, a sensitivity analysis was performed after excluding all borderline tumors (*n* = 86). The analysis therefore included 694 patients, comprising 312 patients with benign tumors and 382 with malignant tumors. The three multivariable logistic regression models were repeated using the same covariates as in the primary analysis. The results remained consistent with the main findings. After adjustment for age and CA-125, higher NLR remained significantly associated with malignancy (OR = 1.91, 95% CI: 1.47–2.48, *p* < 0.001), as did higher PLR (OR = 1.51, 95% CI: 1.20–1.90, *p* < 0.001), whereas higher LMR remained inversely associated with malignant disease (OR = 0.62, 95% CI: 0.47–0.82, *p* < 0.001). These findings indicate that the associations observed in the primary analysis were maintained after exclusion of borderline tumors and were therefore not dependent on their inclusion in the non-malignant reference category.

**Figure 3:**
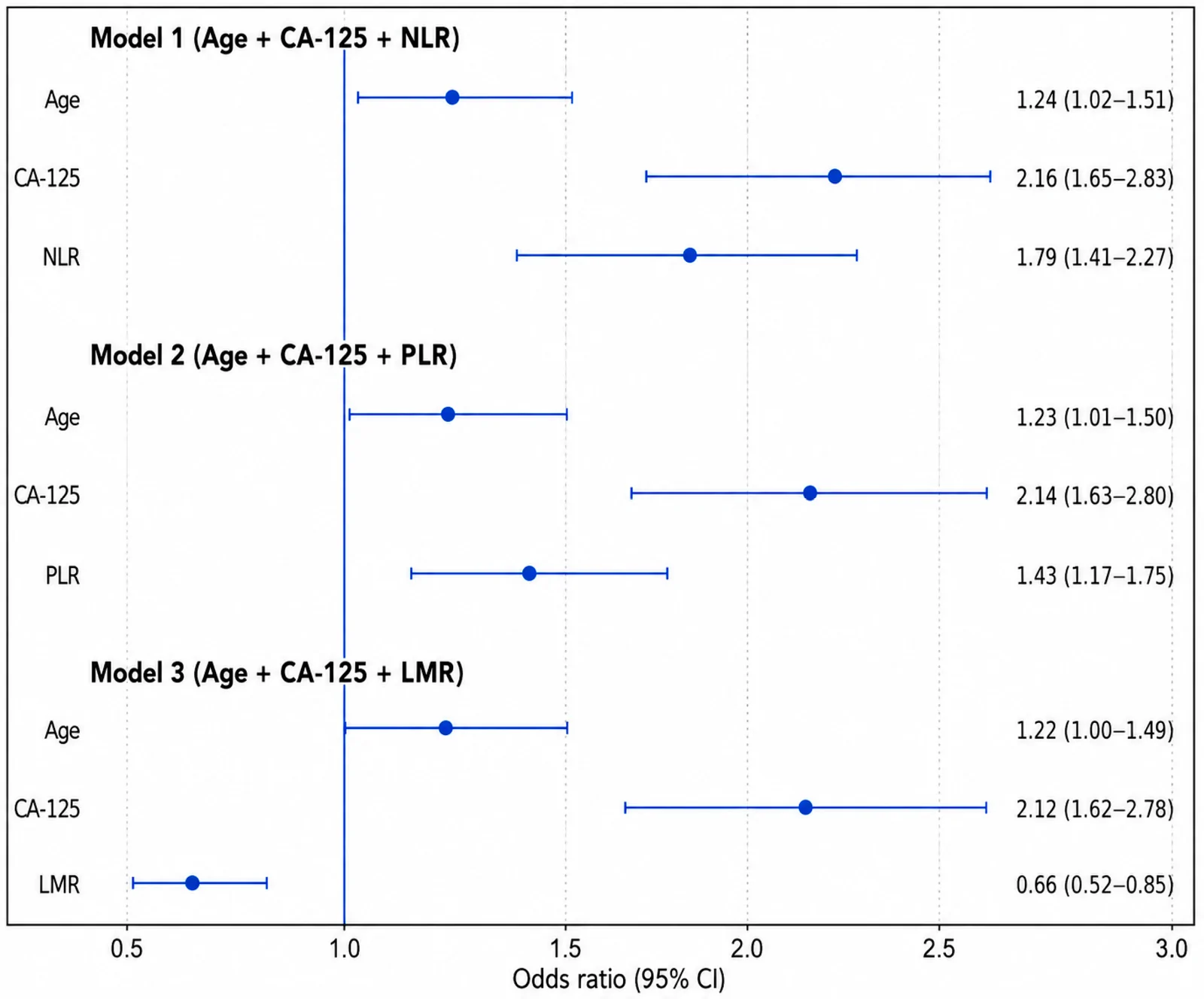
Forest plot summarizing the adjusted associations obtained from three separate multivariable logistic regression models. Model 1 included age, CA-125, and NLR; Model 2 included age, CA-125, and PLR; and Model 3 included age, CA-125, and LMR. Odds ratios (ORs) are presented per one-standard-deviation increase with 95% confidence intervals (CIs). The vertical line at OR = 1 indicates no association.

Across the three separate multivariable models, serum CA-125 remained the strongest predictor of malignant ovarian tumors. As illustrated in Figure 3, across the three separate multivariable models, serum CA-125 remained the strongest predictor of malignant ovarian tumors. Higher NLR and PLR values remained significantly associated with increased odds of malignancy after adjustment for age and CA-125, whereas higher LMR values were inversely associated with malignant disease.

No evidence of poor model fit was identified according to the Hosmer–Lemeshow goodness-of-fit test (all *p* > 0.05), while the models showed comparable explanatory performance, with Nagelkerke’s R^2^ values ranging from 0.43 to 0.47.

**Figure 4:**
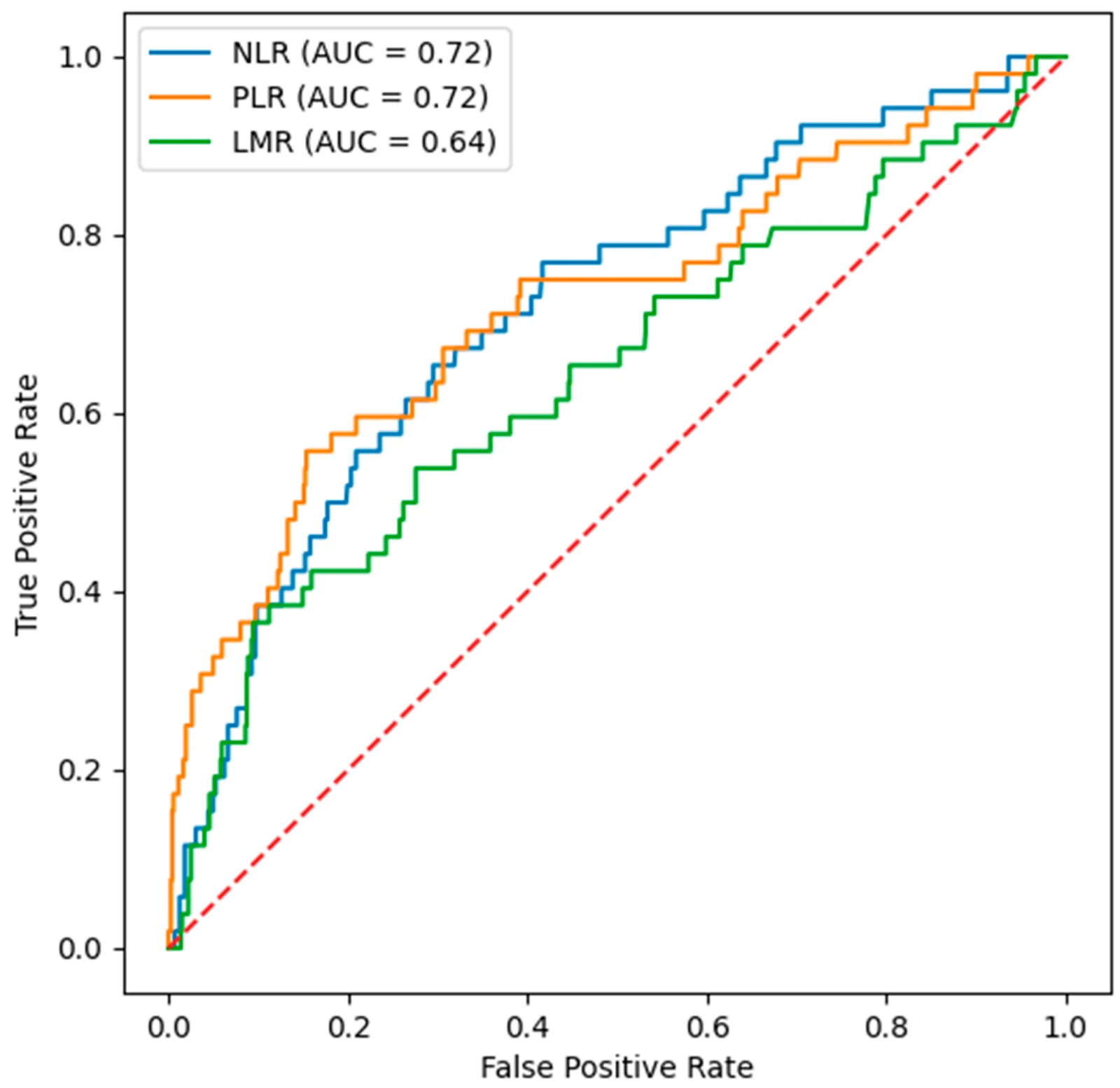
Receiver operating characteristic (ROC) curves illustrating the diagnostic performance of the individual inflammatory indices for predicting malignant ovarian tumors. NLR and PLR demonstrated moderate discriminative ability (AUC = 0.72 for both), whereas LMR showed lower diagnostic performance (AUC = 0.64). The dashed diagonal line represents the line of no discrimination.

The diagnostic performance of the individual inflammatory indices was evaluated using receiver operating characteristic (ROC) curve analysis (Figure 4). CA-125 alone demonstrated good discriminative ability for identifying malignant ovarian tumors (AUC = 0.81). Among the inflammatory indices, NLR and PLR showed moderate discriminative performance (AUC = 0.72 for both), whereas LMR demonstrated lower discriminative ability (AUC = 0.64). The corresponding optimal cutoff values, sensitivities, and specificities for the inflammatory indices are summarized in Table 6. Supplementary analysis, in which the malignant group was restricted to serous, endometrioid, and mucinous epithelial ovarian carcinomas, showed findings consistent with those observed in the overall cohort, with higher NLR and PLR values and lower LMR values remaining associated with malignant disease.

The optimal cutoff values for inflammatory markers were determined using the Youden index and are summarized in Table 6.

For NLR, a cutoff value of 2.74 provided a balanced sensitivity (65.4%) and specificity (70.5%). PLR demonstrated higher specificity (84.6%) at a cutoff of 203.7, albeit with lower sensitivity. LMR showed high specificity (88.8%) but limited sensitivity (38.5%).

The observed diagnostic performance of NLR and PLR supports their potential complementary role as readily available inflammatory biomarkers for the preoperative assessment of ovarian tumors, whereas LMR may provide additional specificity in selected clinical settings.

## 4. Discussion

Overall, the findings of our study are consistent with the existing literature and further support the diagnostic and predictive value of peripheral blood inflammatory markers in ovarian malignancies [26,27,28]. Beyond this general concordance, our results provide additional evidence that simple, routinely available hematological indices may capture clinically relevant aspects of tumor biology and host immune response.

While the association between systemic inflammatory markers and ovarian malignancy has been previously described [26,27,28], the present study provides additional clinically relevant information by evaluating these markers in a comparatively large real-world cohort including benign, borderline, and malignant ovarian tumors. Importantly, the study explored the complementary diagnostic contribution of inflammatory indices beyond CA-125, evaluated each inflammatory index in a separate adjusted multivariable model, and assessed the consistency of the findings within epithelial ovarian carcinoma subgroups. These aspects provide a broader and methodologically robust evaluation of systemic inflammatory markers in ovarian tumor assessment.

From a pathophysiological perspective, the role of neutrophils extends well beyond their classical function in acute inflammation. Molecular studies have demonstrated that neutrophils actively contribute to tumor progression through multiple mechanisms, including activation of the transforming growth factor β (TGF-β) signaling pathway. In addition, they exert immunosuppressive effects by releasing reactive oxygen species and inhibiting cytotoxic lymphocyte activity. In early-stage epithelial ovarian cancer, neutrophil recruitment has been shown to facilitate the formation of a pre-metastatic niche, particularly at the level of the omentum, thereby promoting peritoneal dissemination through epithelial–mesenchymal transition [29,30,31].

NETosis may represent an additional mechanism linking neutrophil activation to ovarian cancer progression. Neutrophil extracellular traps (NETs), released during NETosis, can contribute to a pro-tumor microenvironment by facilitating tumor cell adhesion and metastatic dissemination, while also promoting inflammatory and immunosuppressive signaling within the tumor microenvironment [32].

Consistent with these mechanisms, elevated NLR values were significantly associated with malignant ovarian tumors and remained significantly associated with malignant ovarian tumors in a separate multivariable model adjusted for age and CA-125. This finding reinforces the concept that an imbalance favoring innate inflammatory activity over adaptive immune response is closely linked to tumor aggressiveness.

Similarly, platelets appear to play a central role in all major stages of tumor development, including proliferation, angiogenesis, and metastatic spread. Their involvement is mediated through the release of pro-inflammatory and pro-angiogenic factors such as IL-8 and TGF-β, as well as through interactions with circulating tumor cells and immune components. Moreover, tumor-associated inflammatory signaling, including cytokines such as IL-1, IL-6, and IL-11, can stimulate megakaryopoiesis and thrombopoietin production, ultimately leading to reactive thrombocytosis frequently observed in ovarian cancer patients [33,34].

Our data support these biological observations, demonstrating a progressive increase in PLR across tumor categories. In a separate multivariable model adjusted for age and CA-125, higher PLR remained significantly associated with malignant ovarian tumors. Although its discriminative performance was moderate when considered individually, its adjusted association suggests that PLR may provide complementary diagnostic information in the preoperative assessment of ovarian tumors.

In contrast, lymphocytes represent a fundamental component of the anti-tumor immune response. Through the activity of tumor-infiltrating lymphocytes, particularly CD4+ helper T cells, CD8+ cytotoxic T lymphocytes, and natural killer cells, the host immune system can recognize and eliminate malignant cells. Activation of lymphocytes by antigen-presenting cells leads to the differentiation of cytotoxic effector cells capable of inducing tumor cell apoptosis, while helper T cells enhance this response through cytokine-mediated signaling pathways. However, this anti-tumor effect may be counterbalanced by the presence of immunosuppressive regulatory T cells (Tregs), which inhibit effective immune surveillance [34,35,36,37].

Given these opposing roles, the incorporation of lymphocyte counts into composite inflammatory ratios reflects the dynamic balance between pro-tumor inflammation and anti-tumor immunity [38]. In our cohort, LMR showed an inverse association with malignancy, with lower values observed in malignant tumors. This inverse association remained significant in a separate multivariable model adjusted for age and CA-125. Although LMR demonstrated limited sensitivity, its relatively high specificity suggests that it may provide complementary information in selected clinical settings.

The clinical relevance of CBC-derived inflammatory indices also extends beyond gynecologic oncology. Recent obstetric studies have demonstrated associations between systemic inflammatory indices and clinically relevant pregnancy outcomes. In a prospective observational study, La Verde et al. reported that a composite systemic inflammatory score derived from routinely available hematological indices was inversely associated with birth weight and neonatal head circumference. Similarly, Kırat et al. found significant alterations in several systemic inflammatory indices in women with preterm delivery, with NLR and SIRI remaining independently associated with preterm birth in multivariable analysis. Importantly, the relatively modest individual discriminative performance reported for these indices in obstetric populations further supports their interpretation as complementary markers of systemic inflammatory status rather than standalone diagnostic tools. Together, these findings illustrate the broader biological and clinical relevance of readily available hematological inflammatory indices across different areas of obstetric and gynecologic research [39,40].

An important finding of our study is the consistent contribution of patient age across all adjusted multivariable models. While most previously proposed scores rely exclusively on laboratory parameters [41], our results indicate that age remained significantly associated with ovarian malignancy in all three models after adjustment for CA-125 and one inflammatory index. This observation may reflect the cumulative effects of chronic low-grade inflammation, immunosenescence, and the increasing incidence of malignant ovarian tumors with advancing age [42]. The consistency of the age estimates across the separate NLR, PLR, and LMR models further supports its relevance as an important clinical covariate in preoperative risk assessment. The consistency of these findings suggests that age may represent a useful component of future multivariable risk-stratification approaches without increasing diagnostic complexity [43].

The consistent association of age across all three adjusted models also raises the possibility of incorporating age into future multivariable clinical risk scores together with established biomarkers such as CA-125 and selected inflammatory indices. However, age was analyzed as a continuous standardized variable in the present study, and no age-specific diagnostic threshold was predefined or derived. Therefore, our data do not support proposing a specific age cutoff for clinical decision-making. Future prospective studies should evaluate whether clinically meaningful age thresholds improve discrimination and calibration and whether their incorporation into externally validated risk scores provides incremental value beyond established clinical and biochemical parameters.

Another important observation is that systemic inflammatory markers remained associated with ovarian malignancy after adjustment for CA-125 and age. Although CA-125 remains the most widely used biomarker in ovarian tumor assessment, its diagnostic performance may be limited by suboptimal specificity and variability across histological subtypes. The persistence of the associations observed for NLR, PLR, and LMR in separate adjusted models suggests that the host inflammatory response may provide biological information complementary to that reflected by CA-125. These inflammatory indices should therefore not be interpreted as replacements for CA-125, but rather as complementary biomarkers reflecting distinct aspects of tumor-associated systemic inflammation and host immune response.

The observed heterogeneity in inflammatory profiles may partially reflect differences in tumor histopathology and biological behavior. Epithelial ovarian carcinomas, particularly serous carcinomas, are characterized by a more pronounced inflammatory tumor microenvironment and stronger interaction with systemic immune pathways compared with several non-epithelial ovarian tumors. This may partially explain the consistent associations observed between inflammatory markers and malignant disease in the present cohort. The relatively high proportion of endometrioid carcinomas observed in our cohort (42.9% of malignant tumors) should be interpreted in the context of the single-center design and the specific case mix of a tertiary referral institution. Histopathological classification was based on the definitive diagnoses established following surgical treatment and routine pathological assessment at our institution. Therefore, the distribution of histological subtypes observed in the present cohort primarily reflects the characteristics of the population treated during the study period and should not be considered representative of the expected distribution of ovarian carcinoma subtypes in the general population.

The observed increase in CRP levels among malignant tumors further supports the presence of a more pronounced systemic inflammatory response in malignant disease. However, CRP was not included in the multivariable diagnostic models or ROC analyses in the present study; therefore, no conclusions regarding its independent diagnostic contribution or comparative discriminative performance can be drawn from our data.

From a clinical perspective, the assessment of routinely available inflammatory indices alongside established clinical and laboratory parameters may contribute to preoperative risk stratification, particularly in settings where advanced imaging techniques or specialized biomarkers are not readily available. Nevertheless, the present findings should be interpreted cautiously and require prospective external validation before potential routine clinical implementation [44,45]. Importantly, the diagnostic performance of the inflammatory indices when considered individually was modest. NLR and PLR each yielded an AUC of 0.72, whereas LMR showed lower discrimination (AUC = 0.64), indicating that none of these markers should be interpreted as a standalone diagnostic test for ovarian malignancy. This limitation is also reflected in the cutoff-based performance. At the identified cutoff of 2.74, NLR provided a sensitivity of 65.4% and specificity of 70.5%, while PLR at a cutoff of 203.7 showed a sensitivity of 55.8% and specificity of 84.6%. LMR had the lowest sensitivity (38.5%) at the identified cutoff of 3.15, despite a relatively high specificity of 88.8%. Thus, a substantial proportion of malignant tumors would not be identified if these thresholds were used independently for diagnostic decision-making. Their potential clinical value therefore lies in providing complementary information for preoperative risk stratification alongside established clinical and biochemical parameters, rather than functioning as independent diagnostic tools. Furthermore, these cutoff values were derived and evaluated within the same retrospective cohort and should be regarded as exploratory, cohort-specific thresholds requiring external validation before any clinical application.

Given the retrospective observational design and the absence of longitudinal follow-up data, the present analysis should primarily be interpreted as a diagnostic association study rather than a prognostic cohort investigation.

Although reproductive outcomes were not evaluated in the present study, recent evidence has suggested a possible association between systemic inflammatory markers and ovarian reserve parameters in oncologic patients undergoing fertility preservation procedures [43]. Therefore, the potential relationship between tumor-related systemic inflammation and reproductive biology remains an interesting hypothesis-generating area for future investigation.

Although the observed associations remained statistically significant after adjustment, the present findings should primarily be considered exploratory and hypothesis-generating. The Hosmer–Lemeshow test provided no evidence of poor model fit; however, no formal internal validation procedure, such as bootstrapping or cross-validation, was performed. In addition, more comprehensive calibration assessments, including calibration plots and calibration slope and intercept estimates, and clinical utility analyses such as decision curve analysis were not conducted. Therefore, the reported models should be interpreted as exploratory association models rather than internally validated prediction models. Prospective multicenter studies incorporating formal internal validation and subsequent external validation in independent populations are required before their potential clinical implementation.

Future research should aim to validate these findings in prospective, multicenter cohorts to enhance their external validity and clinical applicability. Incorporating longitudinal follow-up data would allow for a more comprehensive evaluation of the prognostic value of inflammatory markers, particularly in relation to survival outcomes and treatment response.

Further studies could also explore the integration of systemic inflammatory indices into existing clinical and imaging-based risk stratification models, with the goal of developing more accurate and personalized diagnostic algorithms. The addition of molecular and genomic data may further refine these models and improve their predictive performance [46,47,48,49,50].

Another promising direction involves investigating the dynamic changes in inflammatory markers over time, rather than relying solely on baseline values. Such an approach may provide valuable insight into treatment response and disease progression, as longitudinal variations in indices such as NLR and PLR have been shown to correlate with therapeutic outcomes, progression-free survival, and overall prognosis across multiple oncologic settings [51]. Moreover, emerging evidence suggests that temporal biomarker trajectories may offer superior predictive accuracy compared to single time-point measurements, supporting their integration into dynamic predictive models [52,53].

### Strengths, Limitations and Future Directions

The present study has several notable strengths that support the robustness and clinical relevance of its findings. First, the relatively large sample size (*n* = 780) provides adequate statistical power and enhances the reliability of the observed associations between inflammatory markers and ovarian tumor characteristics. The inclusion of a heterogeneous cohort encompassing benign, borderline, and malignant ovarian tumors further allows for a comprehensive evaluation across the entire spectrum of ovarian pathology.

Second, all hematological parameters were obtained prior to any therapeutic intervention, minimizing potential treatment-related confounding and ensuring that the analyzed inflammatory indices reflect the baseline interaction between tumor biology and host immune response. This aspect is particularly important in the context of systemic inflammatory markers, which are highly sensitive to external influences.

Another important strength of the present study is the integration of inflammatory markers with CA-125 and histopathological subgroup analyses, allowing a more comprehensive evaluation of the complementary diagnostic role of systemic inflammation across different ovarian tumor entities.

Another strength lies in the methodological rigor of the statistical analysis. The use of standardized z-scores enabled comparability across variables, while multiple testing corrections, using the Benjamini–Hochberg false discovery rate method, reduced the risk of type I error. In addition, the reporting of 95% confidence intervals provides a more conservative and reliable estimation of effect sizes.

Importantly, an additional methodological strength of the present study is the evaluation of each inflammatory index in a separate multivariable model adjusted for age and CA-125. This approach minimized the risk of collinearity arising from the shared hematological components of NLR, PLR, and LMR while allowing a more reliable assessment of the adjusted association of each inflammatory index with ovarian malignancy. Given that these markers are inexpensive, routinely available, and easily reproducible, they may represent valuable complementary biomarkers for preoperative risk assessment, particularly in resource-limited settings.

Despite these strengths, several limitations should be acknowledged when interpreting the results. The retrospective design inherently introduces the possibility of selection bias and limits the ability to establish causal relationships. In addition, as a single-center study, the findings may not be fully generalizable to other populations with different demographic or clinical characteristics. Another important limitation is the lack of longitudinal survival data. Although the study demonstrates the predictive value of inflammatory markers for malignancy, it does not directly assess their association with long-term oncologic outcomes such as OS or progression-free survival. As such, the term “prognostic” should be interpreted with caution in the absence of survival-based endpoints.

It should be noted that FIGO staging data were not consistently available across all included cases and were therefore not incorporated into the present analysis. This limitation is primarily related to the retrospective design of the study, where data were extracted from routine clinical records that did not uniformly include complete staging information, particularly for patients with benign or borderline tumors, for whom formal FIGO staging is not routinely applied. In addition, variations in documentation practices over the study period further contributed to the heterogeneity of staging data availability. Importantly, the primary objective of the study was to evaluate the predictive value of preoperative systemic inflammatory markers in differentiating tumor type (benign, borderline, malignant), rather than to assess stage-specific outcomes. Consequently, the absence of FIGO staging is unlikely to have significantly influenced the core findings of the analysis. Consequently, stage-stratified analyses could not be performed reliably in the present retrospective cohort and should be addressed in future prospective studies with standardized staging documentation. Nevertheless, the inclusion of standardized staging data in future prospective studies would provide additional insights into the relationship between systemic inflammation and tumor extent.

Although all malignant tumors included in the present study belonged to the epithelial ovarian carcinoma category, benign ovarian tumors demonstrated substantial histopathological heterogeneity, which may have influenced baseline inflammatory profiles.

Although malignant ovarian tumors were analyzed collectively in the primary models, we acknowledge the substantial biological heterogeneity among epithelial ovarian carcinoma subtypes. Serous, endometrioid, and mucinous carcinomas differ in their molecular characteristics, tumor microenvironment, and inflammatory response. Although the supplementary analysis in Table 4 addressed the major epithelial ovarian carcinoma subtypes included in the study, separate subtype-specific diagnostic analyses were not performed. In particular, ROC analyses, AUC estimates, and optimal cutoff values were not calculated individually for serous, endometrioid, and mucinous carcinomas. The unequal distribution of malignant histological subtypes, particularly the relatively small number of mucinous carcinomas (*n* = 36), would have resulted in insufficiently robust estimates for some subtype-specific analyses. Accordingly, we considered it more appropriate to acknowledge this limitation rather than report potentially unstable diagnostic thresholds. Future adequately powered studies should specifically evaluate the diagnostic performance of NLR, PLR, and LMR across individual epithelial ovarian carcinoma subtypes, particularly serous, endometrioid, and mucinous carcinomas. Although separate multivariable models were used to minimize the effects of collinearity, the inflammatory indices evaluated in the present study remain derived from overlapping hematological parameters. Therefore, these biomarkers should be interpreted as providing complementary rather than interchangeable information regarding ovarian malignancy, and their diagnostic utility requires confirmation in independent external validation cohorts.

Residual confounding should also be considered when interpreting the observed associations. Although predefined exclusion criteria were applied to minimize the influence of major conditions known to alter systemic inflammatory markers, including active infection, chronic inflammatory or autoimmune diseases, hematological disorders, and relevant treatments, the retrospective nature of the study did not allow all potential clinical factors affecting leukocyte and platelet counts to be systematically identified or controlled. Variables such as body mass index, smoking status, menopausal status, medication use, and the complete spectrum of medical comorbidities were not consistently available in the medical records. Therefore, unmeasured or undocumented clinical factors may have influenced the observed inflammatory profiles, and residual confounding cannot be completely excluded.

## 5. Conclusions

The present study found significant associations between preoperative systemic inflammatory indices and ovarian tumor malignancy. Higher NLR and PLR values and lower LMR values remained associated with malignant disease in separate multivariable models adjusted for age and CA-125, suggesting that these routinely available indices may provide complementary information beyond established clinical and biochemical parameters. However, their individual discriminative performance was modest, with AUCs of 0.72 for NLR and PLR and 0.64 for LMR, and the identified cutoffs showed important limitations in sensitivity, particularly for PLR (55.8%) and LMR (38.5%). Therefore, these markers should not be interpreted as standalone diagnostic tests, but rather as potential complementary tools for preoperative risk stratification alongside CA-125 and other established parameters. Given the retrospective, single-center design, the possibility of residual confounding, and the absence of formal internal and external validation, these findings should be considered exploratory. Prospective, adequately powered, multicenter studies with independent external validation are required before these inflammatory indices or their derived thresholds can be recommended for routine clinical decision-making. Future validation should preferably be conducted in a prospective, multicenter cohort with an independently recruited population and adequate representation of benign, borderline, and malignant ovarian tumors. The required sample size should be determined a priori through formal power calculations based on the expected discrimination and prevalence of malignancy, with sufficient numbers to permit both overall model validation and clinically relevant subgroup analyses.

## Figures and Tables

**Table 1:** Baseline demographic and laboratory characteristics of the study population.

Variable	Overall Cohort
Total number of patients, *n*	780
Age, years ^(a)^	42.9 ± 15.0
Age ^(b)^	42.0 (31.0–52.0)
Complete blood count parameters at diagnosis	
Leukocytes *	7.99 ± 2.82
Neutrophils *	5.17 ± 2.57
Lymphocytes *	2.19 ± 0.70
Monocytes *	0.44 ± 0.23
Platelets *	312.63 ± 100.13
Inflammatory indices	
NLR ^(a)^	2.66 ± 2.09
NLR ^(b)^	2.14 (1.49–3.09)
PLR ^(a)^	157.45 ± 76.91
PLR ^(b)^	137.84 (110.53–183.21)
LMR ^(a)^	6.01 ± 3.32
LMR ^(b)^	5.36 (4.03–7.09)
Other laboratory parameters	
C-reactive protein (mg/L) ^(a)^	27.15 ± 54.73
CRP ^(b)^	8.6 (6.0–18.0)
CA-125 * ^(a)^	171.89 ± 532.08
CA-125 ^(b)^	21.9 (13.3–85.9)

Continuous variables are presented as mean ± standard deviation ^(a)^ and median (interquartile range, IQR) ^(b)^, as appropriate. * Hematological parameters are expressed as ×10^3^/µL. NLR: neutrophil-to-lymphocyte ratio; PLR: platelet-to-lymphocyte ratio; LMR: lymphocyte-to-monocyte ratio; CRP: C-reactive protein; CA-125: cancer antigen 125.

**Table 2:** Comparison of clinical and inflammatory parameters according to tumor type.

Variable	Benign (*n* = 312)	Borderline (*n* = 86)	Malignant (*n* = 382)	*p*-Value
Age, years ^(a)^	39.8 ± 14.2	44.6 ± 13.8	45.8 ± 15.4	<0.001
NLR ^(b)^	2.12 (1.45–2.86)	2.41 (1.63–3.12)	3.01 (1.98–4.26)	<0.001
PLR ^(b)^	128.5 (102.3–162.7)	142.8 (110.5–178.2)	172.9 (135.4–221.6)	<0.001
LMR ^(b)^	6.48 (4.92–8.11)	5.89 (4.35–7.22)	4.72 (3.61–6.08)	<0.001
Leukocytes * ^(a)^	7.54 ± 2.41	7.88 ± 2.63	8.41 ± 3.07	0.002
Neutrophils * ^(a)^	4.62 ± 2.08	4.98 ± 2.32	5.83 ± 2.91	<0.001
Lymphocytes * ^(a)^	2.32 ± 0.68	2.21 ± 0.66	2.05 ± 0.71	0.004
Platelets * ^(a)^	288.6 ± 85.4	305.2 ± 94.3	335.9 ± 110.7	<0.001
CRP (mg/L) ^(b)^	6.1 (4.0–10.8)	9.4 (6.1–17.6)	18.9 (8.7–41.5)	<0.001
CA-125 ^(b)^	18.5 (10.2–35.6)	42.7 (18.9–96.4)	186.3 (54.2–642.5)	<0.001

Continuous variables are presented as mean ± standard deviation ^(a)^ or median (interquartile range, IQR) ^(b)^, as appropriate. Group comparisons for age, leukocyte, neutrophil, lymphocyte, and platelet counts were performed using one-way ANOVA, whereas NLR, PLR, LMR, CRP, and CA-125 were compared using the Kruskal–Wallis test. * Hematological parameters are expressed as ×10^3^/µL. NLR—neutrophil-to-lymphocyte ratio; PLR—platelet-to-lymphocyte ratio; LMR—lymphocyte-to-monocyte ratio; CA-125—cancer antigen 125.

**Table 5:** Separate multivariable logistic regression models evaluating inflammatory indices as predictors of malignant ovarian tumors.

Variable	SD Used for Standardization	OR (95% CI)	*p*-Value
Model 1 (Age + CA-125 + NLR)			
Age	15.0 years	1.24 (1.02–1.51)	0.018
CA-125	532.08 U/mL	2.16 (1.65–2.83)	<0.001
NLR	2.09	1.79 (1.41–2.27)	<0.001
Model 2 (Age + CA-125 + PLR)			
Age	15.0 years	1.23 (1.01–1.50)	0.021
CA-125	532.08 U/mL	2.14 (1.63–2.80)	<0.001
PLR	76.91	1.43 (1.17–1.75)	0.001
Model 3 (Age + CA-125 + LMR)			
Age	15.0 years	1.22 (1.00–1.49)	0.024
CA-125	532.08 U/mL	2.12 (1.62–2.78)	<0.001
LMR	3.32	0.66 (0.52–0.85)	0.001

Odds ratios are expressed per one-standard-deviation increase in each continuous variable. The SD values used for standardization are reported to facilitate clinical interpretation of the effect estimates.

**Table 6:** Diagnostic performance of individual inflammatory indices for predicting malignant ovarian tumors.

Marker	Cutoff	Sensitivity (%)	Specificity (%)	AUC
NLR	2.74	65.4	70.5	0.72
PLR	203.7	55.8	84.6	0.72
LMR	3.15	38.5	88.8	0.64

Optimal cutoff values were determined using the Youden index. AUC: area under the receiver operating characteristic (ROC) curve.

## Data Availability

The data presented in this study are available upon request from the corresponding author. The data are not publicly available due to hospital policy.
